# Association Between Parental Social Position and Childhood Overweight: Mediation by Lifestyle and BMI Patterns During Pregnancy

**DOI:** 10.1111/ijpo.70047

**Published:** 2025-08-05

**Authors:** Camille Le Gal, Mireille C. Schipper, Marion Lecorguillé, Laura Pavicic, Thierry Simeon, Marie‐Aline Charles, Romy Gaillard, Sandrine Lioret, Barbara Heude

**Affiliations:** ^1^ Université Paris Cité and Université Sorbonne Paris Nord Paris France; ^2^ Generation R Study Group, Erasmus MC University Medical Center Rotterdam the Netherlands; ^3^ Department of Pediatrics, Sophia's Children's Hospital Erasmus MC, University Medical Center Rotterdam the Netherlands; ^4^ Ums INED‐INSERM‐EFS Paris France

**Keywords:** childhood overweight, migration, prenatal lifestyle factors, social inequalities

## Abstract

In high‐income countries, children born to parents with low socio‐economic position (SEP) or with non‐Western ethnicity are disproportionally affected by obesity as early as preschool age. We assessed how much of these associations were mediated by parental lifestyle and BMI patterns during pregnancy. We characterised 5–6 years old children with or without overweight from the French Etude Longitudinale Française depuis l'Enfance (ELFE) (*n* = 8584) and the Dutch Generation R birth cohorts (*n* = 6511). We used counterfactual mediation analyses to assess the potential mediating effect of previously identified lifestyle patterns: “high parental smoking, poor‐quality maternal diet and sedentary behaviour” and “high parental body mass index and low gestational weight gain”. Both patterns jointly mediated 62.8% of the association between parents' education level and childhood overweight in ELFE and 23.2% in Generation R. In Generation R, they jointly mediated 8.9% of the association between parents' geographic origin and childhood overweight. In ELFE, parents with non‐Western backgrounds were less likely to follow the first pattern, resulting in a negative indirect effect. Parental lifestyle and BMI patterns during pregnancy seem key contributors to the early development of socio‐economic inequalities in childhood overweight, while other yet unidentified factors may contribute to inequalities related to geographic origin.

## 1 Introduction

A recent Non‐Communicable Disease Risk Factor Collaboration and WHO study, analysing data for 63 million individuals aged 5 to 19 years across 190 countries, revealed an increase in childhood and adolescent obesity from 31 million in 1990 to 160 million in 2022 [1, 2]. For European children aged 2 to 7 years, the prevalence of overweight (obesity included) was estimated at 17.9% in 2016 [3]. Multiple studies [4, 5] have demonstrated a negative link between parent's socio‐economic position (SEP) and risk of childhood overweight early in life. Early overweight predisposes children to a multitude of comorbidities [6] and also substantially increases their risk of adult obesity [7], thereby reinforcing socio‐economic health inequalities throughout life [8].

Besides SEP, evidence across Europe suggests that children from parents with an immigrant background may be at increased risk of overweight as compared with their native counterparts [9, 10, 11]. Our previous study [12] of the French Etude Longitudinale Française depuis l'Enfance (ELFE) birth cohort confirmed that children of immigrant mothers, and to a lesser extent those born to descendants of immigrants, were at an increased risk of overweight from as early as age 3.5 years as compared with their peers born to non‐immigrant mothers. Likewise, work by Van Rossem et al. [13] identified a higher obesity risk trajectory among children of non‐Dutch ethnic background in the Generation R birth cohort, in The Netherlands up to age 4 years.

Such an early social patterning of overweight is a major public health concern but also a matter of social justice. A better understanding of the mechanisms involved is needed to facilitate more effective efforts to address this injustice, but these causal pathways remain underexplored. This gap in knowledge becomes particularly critical considering the notable prevalence of overweight in very young children, which underscores the importance of early life risk factors. The preconception and pregnancy periods are increasingly recognised as a window of vulnerability for the programming of cardiometabolic health [14]. In the literature, individual risk factors identified with a robust level of evidence include high maternal body mass index (BMI), excessive gestational weight gain, unhealthy diet, smoking, and sedentary behaviours [15, 16]. These lifestyle and BMI‐related factors are also socially patterned [17, 18]. However, they are often analysed separately even though they interact with each other and combine into patterns, with supposedly synergistic effects on both mother and child health. Furthermore, these lifestyle behaviours and BMI status are related between parents. In the context of the European EndObesity consortium, Lecorguillé et al. [19] used an integrative approach to synthesise mothers' and fathers' lifestyle and BMI factors during pregnancy into patterns. Two of these patterns were common to the ELFE and Generation R cohorts and were found associated with increased risk of childhood overweight. The first lifestyle pattern was characterised by high parental smoking, poor‐quality maternal diet, and sedentary behaviour in the French ELFE cohort and high parental smoking and poor‐quality maternal diet in the Dutch Generation R cohort. The second pattern featured high parental body mass index and low gestational weight gain.

Building on these novel insights, along with the known social patterning of lifestyle behaviours and BMI in adults [20], the current study investigated to what extent these parental lifestyle and BMI patterns during pregnancy mediate the association between parental social position and their child's risk of overweight. Social position was assessed using multiple dimensions: parents' geographic origin and parents' SEP, defined by education level and household income. We tested this mediation hypothesis in two distinct European populations, the ELFE and Generation R cohorts, to allow for comparing results between different settings.

## 2 Materials and Methods

### 2.1 Study Population

The multidisciplinary and national ELFE birth cohort [21] enrolled 18 329 children from a random sample of 344 maternity units across hexagonal France in 2011. The enrollment took place over 25 days in four waves of 4 to 8 days each, covering all four seasons. Inclusion criteria were single or twin births ≥ 33 weeks of gestation and mothers aged ≥ 18 years and not planning to leave mainland France for the next 3 years. Recognising the language diversity among the population, study materials, including the information letter and consent form, were made available in Arabic, Turkish, and English, the three most commonly spoken languages in France among non‐French speakers. Consent for their child's participation was obtained from 51% of eligible mothers, with fathers (if present) given the chance to consent and informed of their right to oppose the study.

The Generation R study is a prospective cohort study from early pregnancy onward in Rotterdam, The Netherlands [22]. Eligible mothers had to live in the study area with a delivery date from April 2002 to January 2006. The study aimed to enrol mothers in early pregnancy (gestational age < 18 weeks), but enrolment was allowed until the birth of the child. In total, 9778 mothers were included in the study (response rate 61%), 91% (*n* = 8880) during pregnancy. Fathers were invited to participate, with 71% (*n* = 6347) enrolling. Mothers and fathers were asked for their written informed consent. At the start of each phase, mothers and fathers received written and oral information about the study.

### 2.2 Data Collection and Measurements

For analyses, preference was given to using variables that were harmonised [23] between the two cohorts from previous work conducted as part of the EU Child cohort Network which operates as a federated analysis infrastructure with harmonised datasets [24].

### 2.3 Social Position (Exposure): Geographic Origin and Socio‐Economic Position (Education Level and Income)

In the ELFE cohort, maternal and paternal geographic origin data and SEP factors, including education level and household income, were collected by computer‐assisted telephone interviews when the child was 2 months old. In the Generation R cohort, all three factors were collected with self‐reported questionnaires at study enrollment.

The child's geographic origin was classified as “at least one non‐Western parent” versus “no non‐Western parent”. Parents' education was determined by the highest level achieved by either of the child's parents and classified as “≤ high school degree” versus “≥ undergraduate degree” (corresponding to short‐cycle tertiary education or above). Household income was dichotomized according to the first quintile of its distribution in ELFE and based on 1200 €/monthly in Generation R. These definitions are further detailed in Supporting Information.

### 2.4 Parental Lifestyle and BMI Patterns (Candidate Mediators)

The two parental lifestyle and BMI patterns from the previously mentioned work by Lecorguillé et al. [19] were derived by using principal component analyses and thus are independent (orthogonal) by construction. Each cohort mother–father dyad has a score on each lifestyle pattern, with high (low) scores indicating high (low) adherence to a given pattern [19].

### 2.5 Childhood Anthropometrics (Outcome)

Anthropometric measurements were collected by telephone when the child was about age 5 in ELFE: parents were asked to report their child's weight and height as recorded in the health booklet. In Generation R, weight and height were measured in all children at about 6 years at the research centre. Child BMI was calculated as weight (kg)/[height (m)]^2^. Children were classified as having overweight (including obesity) or thin/normal BMI status based on age‐ and sex‐specific cut‐offs from the International Obesity Task Force (IOTF), corresponding to an adult BMI equivalent of ≥ 25 kg/m^2^.

### 2.6 Covariates

Covariates, including child sex, maternal age at delivery, and parity, were collected by in‐person interviews upon delivery at the maternity hospital in ELFE, and details on maternal health history were collected from medical records. In Generation R, covariates were obtained from medical records or by questionnaires at enrollment or during pregnancy, as described in the statistical analysis section.

### 2.7 Study Sample Selected for Statistical Analysis

**FIGURE 1:**
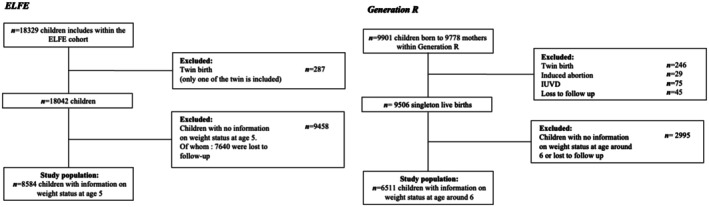
Flow chart of the study sample in the ELFE (left) and Generation R (right) cohort.

In ELFE, we included one randomly selected twin, whereas in Generation R, twins were excluded from the analysis because the proportion of missing data was higher in this group and imputation was questionable because of the inherent specificities of this population (Figure 1). Anthropometric measurements among children were selected from age ranges of 4.2 to 6.5 years (average 5.0 years) in ELFE and 4.8 to 9.1 years (average 6.2 years) in Generation R. Only children with at least one BMI measure available within these age ranges in both cohorts were eligible for analyses (*N* = 8584 in ELFE and *N* = 6511 in Generation R). When multiple BMI measures were available for the same child, only the last one was used.

### 2.8 Statistical Analysis

**FIGURE 2:**
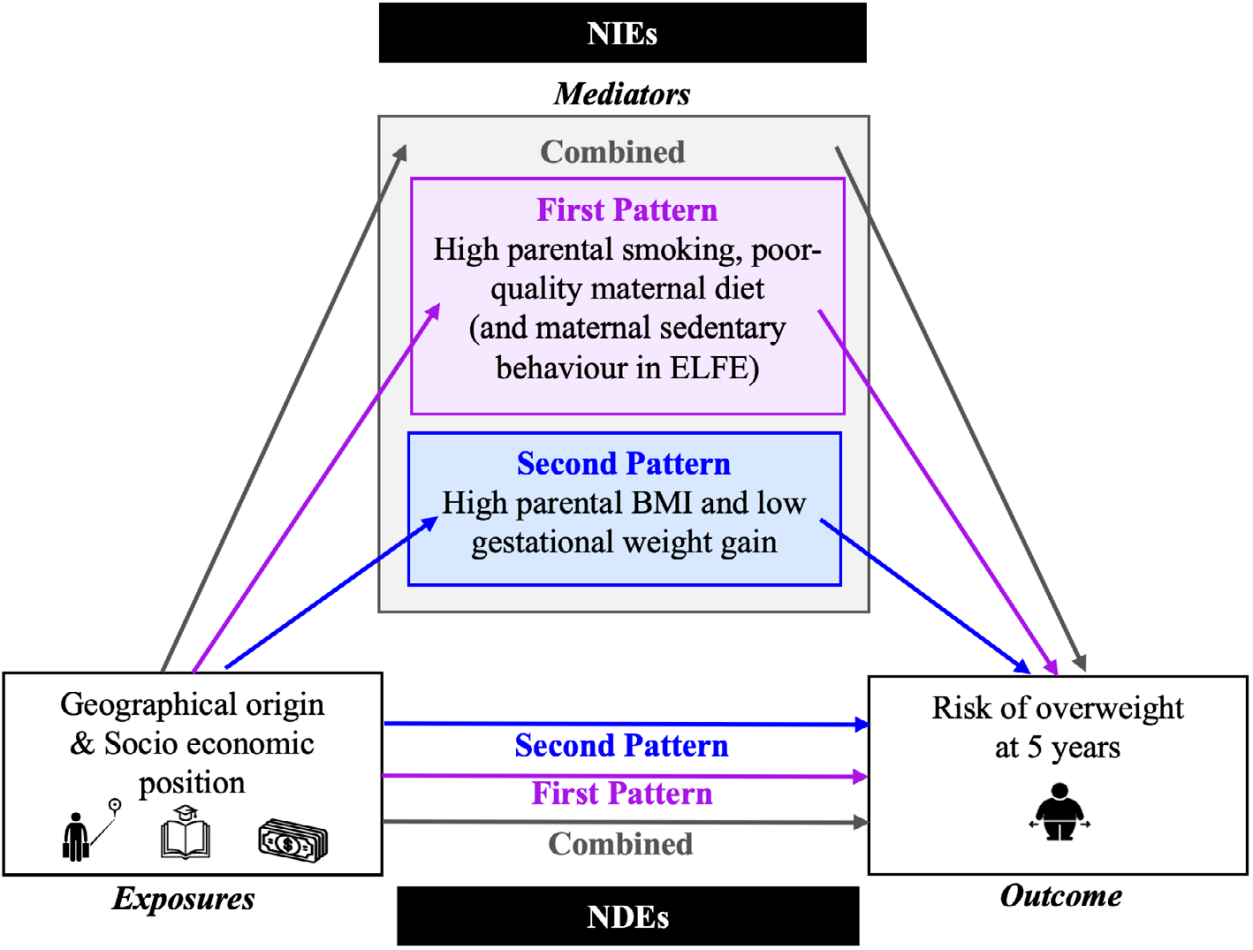
Conceptual framework for mediation analyses. Baseline assessment during pregnancy.

We examined the associations between indicators of social position and the two previously derived parental lifestyle and BMI patterns (derived by Lecorguillé et al. [19]) using multivariable linear regression analyses. We used counterfactual mediation analyses to explore the role of these patterns in the causal pathway between social position indicators and the risk of overweight in children at ages 5 to 6, analysing each cohort separately. Counterfactual mediation analysis was used to break down the total effect of each social position indicator under study on child's overweight risk into the sum of a natural direct effect (NDE, independent of the candidate mediators) and natural indirect effect (NIE, via candidate mediators) (Figure 2). As an example, for parents' education level, the total effect can be considered as the change in overweight risk if the education level changed from “≥ undergraduate degree” to “≤ high school degree”. Here, the NDE would be the change in overweight risk from the same shift in education level while keeping mediators at the values they naturally take when the education level is unchanged (e.g., “≥ undergraduate degree”). Also, the NIE would be the change in overweight risk if parents' education level remained unmodified (e.g., “≥ undergraduate degree”) but mediators changed to the values they would naturally take if the education level had shifted from “≥ undergraduate degree” to “≤ high school degree”. For these analyses, we used the Medflex package in RStudio (2022) (RStudio: Integrated Development Environment for R. RStudio, PBC, Boston, MA) dedicated to counterfactual mediation analysis. This allowed us to estimate the mediated proportion attributable to each lifestyle and BMI pattern individually as well as the collective mediated effect of the two patterns combined. Of note, the proportion could not be estimated and was therefore not reported when the natural direct and indirect effects were of opposite signs.

All association and mediation analyses were adjusted for child sex, maternal age at delivery, and parity. Analyses focusing on parents' education level were further adjusted for parents' geographic origin. The examined income levels were adjusted for both parents' geographic origin and education level. These adjustments were consistent with the previous work by Le Gal et al. [12], which structured analyses following a socio‐ecological framework. We assumed that parents' geographic origin and education level were at the most distal levels, with the latter influencing income level, the most proximal variable. This hierarchical approach was designed to ensure that intermediate exposures did not affect the association of the distal exposures with the outcome under study [25, 26].

We used the Multivariate Imputation by Chained Equations [27] package in R to address missing data, including all relevant variables providing information for the imputation process and assuming the data were missing at random. We generated 10 imputed datasets and ensured reproducibility by using a fixed random seed. The results of the imputed datasets were combined using the MIcombine function in R, which estimates combined effects and their confidence intervals. This method aligns with Rubin's rules [28] by considering both within‐ and between‐imputation variability, ensuring the standard errors reflect the uncertainty due to missing data.

In accordance with the STROBE (Strengthening the Reporting of Observational Studies in Epidemiology) guidelines, we adhered to its methodological framework and provided a detailed checklist in the Supporting Information.

## 3 Results

In the ELFE and Generation R cohorts, 696 of 8594 (8.1%) and 1149 of 6511 (17.6%) children included in the study samples exhibited overweight at age 5 to 6 years (Table 1). Notably, the proportion of non‐Western parents was threefold higher in Generation R than in ELFE (37.9% vs. 12.3%). In addition, the proportion of parents with an education level below a high school degree was twice as high in Generation R than in ELFE (50.3% vs. 23.2%).

**TABLE 1:** Characteristics of the ELFE (*N* = 8584) and Generation R (*N* = 6511) samples at age 5 to 6 years.

	ELFE (*N* = 8584)	Generation R (*N* = 6511)
Child with overweight	8.1% (696)	17.6% (1149)
Sex of the child		
Girl	48.5% (4162)	49.8% (3240)
Maternal age at delivery (years), Mean (SD)	30.90 (4.59)	31.0 (5.20)
Parity		
First time mother	46.9% (3978)	54.6% (3554)
Second time mother	36.5% (3096)	29.3% (1908)
≥ Third time mother	12.3% (1045)	12.8% (832)
Missing values	102	217
Parent's geographical origin		
At least one Non‐Western parent	12.3% (1054)	37.9% (2468)
Missing values	255	176
Parent's education level		
≤ High school degree	23.2% (1992)	50.3% (3272)
Missing values	0	0
Household income* (€)		
1st Quintile or ≤ 1200 €	12.4% (1063)	13.0% (844)
Missing values	452	1601

*Note:* As total household income in the Generation R cohort.

* Household income is expressed per consumption unit in the ELFE cohort, whereas it is reported.

In ELFE, parents with non‐Western backgrounds were less likely to adhere to the first pattern, characterised by high parental smoking, poor‐quality maternal diet, and maternal sedentary behaviour than their Western counterparts; no such association was observed in Generation R (Table 2). SEP factors were inversely associated with this lifestyle pattern in both cohorts. Parents from non‐Western backgrounds, with a low education level (in both cohorts), or those with a low income (in ELFE only) were more likely to follow the second pattern, characterised by high parental BMI and low gestational weight gain; of note, parents with a low income had lower scores on this pattern in Generation R than ELFE.

**TABLE 2:** Association between parental social position indicators and parental lifestyle and BMI patterns in ELFE and Generation R cohorts.

	High parental smoking, poor‐quality maternal diet (and maternal sedentary behaviour in ELFE)								High parental BMI and low gestational weight gain					
	ELFE				Generation R				ELFE			Generation R		
	*N*	*β*	95% CI	*p*	*N*	*β*	95% CI	*p*	*β*	95% CI	*p*	*β*	95% CI	*p*
Parents' geographic origin	8229			< 0.001	5917			0.5			0.002			< 0.001
No Non‐Western parent		—	—			—	—		—	—		—	—	
At least one Non‐Western parent		−0.21	−0.29; −0.13			0.03	−0.05; 0.11		−0.11	−0.04; −0.19		−0.28	−0.34; −0.21	
Parents' education level	8229			< 0.001	5917			< 0.001			0.008			< 0.001
≥ Undergraduate degree		—	—			—	—		—	—		—	—	
≤ High school degree		0.84	0.78; 0.90			0.71	0.63; 0.79		−0.08	−0.02; −0.14		−0.19	−0.26; −0.13	
Household income* (€)	7985			< 0.001	4803			< 0.001			< 0.001			0.012
> 1st Quintile or > 1200 €		—	—			—	—		—	—		—	—	
1st Quintile or ≤ 1200 €		0.21	0.13; 0.29			0.32	0.20; 0.45		−0.18	−0.11; −0.26		0.13	0.03; 0.23	

*Note:* As total household income in the Generation R cohort. All analyses were adjusted for child sex, maternal age at birth, and parity. Additionally, analyses focusing on parents' education level were further adjusted for parent's geographical origin. Those examining income level were adjusted for both parent's geographical origin and parent's educational level.

Abbreviations: BMI, body mass index; CI, Confidence interval.

* Household income is expressed per consumption unit in the ELFE cohort, whereas it is reported.

**FIGURE 3:**
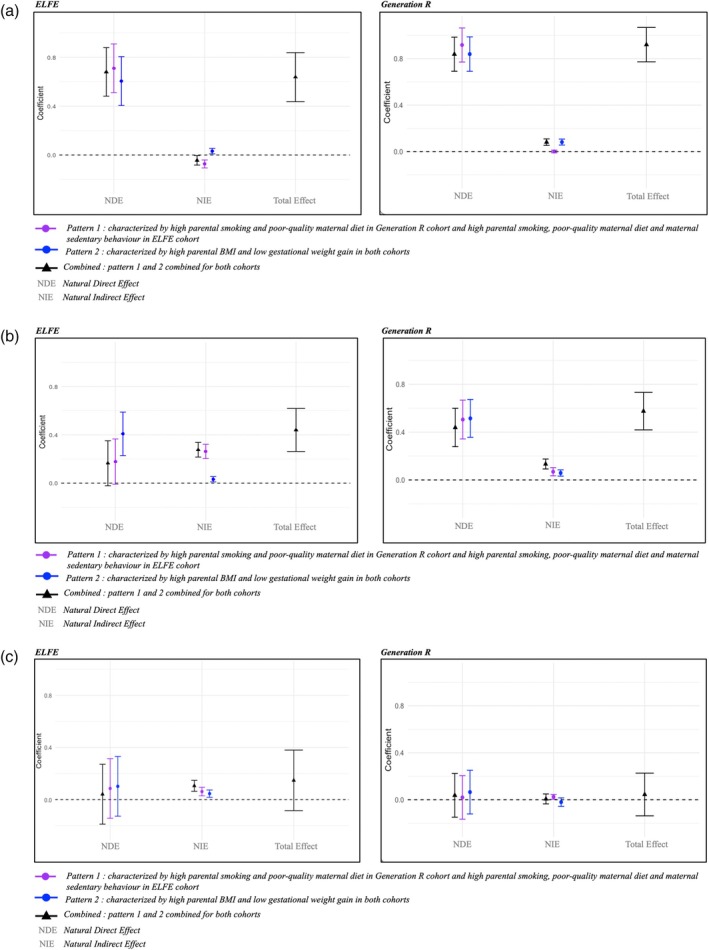
(a) Mediating effect of parental lifestyle and BMI patterns during pregnancy on the association between parents' geographic origin and risk of overweight in children: Results at age 5 to 6 years from the ELFE cohort and Generation R cohort. Here the total effect can be considered as the change in overweight risk if parents' geographic origin changed from “no Non‐Western parent” to “at least one non‐Western parent”. The NDE would be the change in overweight risk from the same shift in geographic origin while keeping mediators at the values they naturally take when geographical origin is unchanged (e.g., “no Non‐Western parent”). Also, the NIE would be the change in overweight risk if geographic origin remained unmodified (e.g., “no Non‐Western parent”) but mediators changed to the values they would naturally take if geographic origin had shifted from “no Non‐Western parent” to “at least one non‐Western parent”. Analyses performed on imputed data and adjusted for child's sex, maternal age, and parity. (b) Mediating effect of parental lifestyle and BMI patterns during pregnancy on the association between parents' education level and risk of overweight in children: Results at age 5 to 6 years from the ELFE cohort and Generation R cohort. Analyses performed on imputed data and adjusted for child's sex, maternal age, parity, and parents' geographic origin. Counterfactual hypothesis: Here the total effect can be considered as the change in overweight risk if parents' education level changed from “≥ undergraduate degree” to “≤ high school degree”. The NDE would be the change in overweight risk from the same shift in education level while keeping mediators at the values they naturally take when education is unchanged (e.g., “≥ undergraduate degree”). Also, the NIE would be the change in overweight risk if parents' education level remained unmodified (e.g., “≥ undergraduate degree”) but mediators changed to the values they would naturally take if parents' education level had shifted from “≥ undergraduate degree” to “≤ high school degree”. (c) Mediating effect of parental lifestyle and BMI patterns during pregnancy on the association between household income level and risk of overweight in children: Results at age 5 to 6 years from the ELFE cohort and Generation R cohort. Analyses performed on imputed data and adjusted for the child's sex, maternal age, parity, parents' geographic origin, and parents' education level. Counterfactual hypothesis: Here the total effect can be considered as the change in overweight risk if household income level changed from “high and medium” to “low”. The NDE would be the change in overweight risk from the same shift in income while keeping mediators at the values they naturally take when income is unchanged (e.g., “high and medium”). Also, the NIE would be the change in overweight risk if household income remained unmodified (e.g., “high and medium”) but mediators changed to the values they would naturally take if household income level had shifted from “high and medium” to “low”. *“High and medium” categories correspond here to “Quintile > 1 (ELFE) or > 1200 euros (Generation R)” and “low” category corresponds to “1st Quintile (ELFE) or ≤ 1200 € (Generation R)”.

When considering the association between parents' geographic origin and child overweight, the NIE for the combined parental lifestyle and BMI patterns in ELFE was negative (β_NIE_ −0.04 [95% CI −0.08; −0.01]) (Figure 3a). When analysed separately, only the first lifestyle pattern had a negative NIE (β_NIE_ −0.07 [−0.11; −0.04]). The second pattern positively mediated a small portion of the association (β_NIE_ 0.03 [0.01; 0.05]). In Generation R, the NIE for the combined patterns was positive (β_NIE_ 0.08 [0.05; 0.11]), corresponding to a mediation of 8.9% of the total effect. The main contributor was the second pattern, accounting for almost the entire mediation effect.

Regarding SEP, the two lifestyle and BMI patterns combined explained 62.8% of the association between parents' education level and childhood overweight (β_NIE_ 0.27 [0.22; 0.34]) in ELFE (Figure 3b). The first pattern contributed more than the second one (β_NIE_ 0.26 [0.20; 0.32]) versus (β_NIE_ 0.03 [0.01; 0.05]). In Generation R, the global mediating effect was 23.2% (β_NIE_ 0.13 [0.09; 0.17]), with both parental lifestyle and BMI patterns contributing almost equally.

Although the total effect of household income level on childhood overweight was close to zero in both cohorts after adjusting for parents' geographic origin and education level, the two patterns combined mediated a major proportion of the association in ELFE: 71.8% (β_NIE_ 0.10 [0.06; 0.15]) (Figure 3c). We found no mediating effect for this association in the Generation R cohort.

## 4 Discussion

This study is among the first to explore early mechanisms contributing to social inequalities in childhood overweight, by addressing different dimensions of social position along with the lifestyle and BMI status of both parents during pregnancy. In two birth cohorts from France and The Netherlands, common lifestyle and BMI patterns mediated an important part of the association between SEP and childhood overweight. Yet, these patterns had inconsistent mediating effects across the cohorts for the association between parents' geographic origin and childhood overweight, showing a negative indirect effect in ELFE but not Generation R.

To the best of our knowledge, no other study has assessed how parental lifestyle and BMI patterns during pregnancy mediate the association of parents' geographic origin and child overweight, so direct comparisons with the literature are challenging. Our prior research in ELFE [12] showed that the increased likelihood of overweight in 3.5‐year‐old children born to mothers with an immigration background was only slightly mitigated after adjusting for maternal education level, maternal occupational category, and household income level. Likewise, in the Generation R cohort, Van Rossem et al. [13] found that the increased likelihood of overweight in 2‐year‐old children born to Turkish or Moroccan parents versus those born to native Dutch parents was only slightly mitigated after adjusting for maternal education level, household income, and material hardship. Altogether, the results of these two studies suggest that the influence of parental migration status/ethnicity on childhood overweight is mainly due to factors other than these dimensions of SEP.

The differences in mediation effects we observed between cohorts is likely due to substantial socio‐demographic disparities. As compared with the ELFE cohort, the Generation R cohort has a higher proportion of parents born outside the cohort's country. Their non‐Western population also differs in nationalities, with immigrants originating mainly from Turkey, Morocco, and Suriname in The Netherlands and from Maghreb and Sub‐Saharan Africa in France. Another explanation could also partly derive from the theory of the healthy migrant effect: individuals capable of migrating generally have higher economic resources, better health status, and healthier lifestyle on average than their non‐migrating counterparts, both in their country of origin and in the host country [29]. However, this paradigm might vary depending on the specific geographic origin, host country, and reasons for migration, which differ by context [30]. Previous findings from the ELFE study showed that immigrant mothers have healthier dietary habits regardless of their SEP [31] and a lower smoking prevalence as compared with non‐immigrant mothers [32]. Also, smoking prevalence was found to be low among non‐Western populations in The Netherlands [33].

However, these explanations do not elucidate the mechanisms underlying the total effect of geographic origin on childhood overweight. Beyond SEP dimensions, unmeasured social determinants of health, such as language proficiency, health literacy, social support, healthcare access, and other psychosocial factors may also play a role [34, 35, 36]. Especially, factors related to stigma or structural discrimination can lead to chronic psychosocial stress, identified as a contributor to the social origin of obesity [35, 37, 38] Another likely mediation hypothesis involves parenting practices related to the child's diet and movement behaviours early in life, the confirmation of which was beyond the scope of the study: in particular, immigrant parents may face specific barriers to maintaining their traditional lifestyle for their child because of conflicting social norms and expectations in their host country [39, 40].

For mediation analyses involving parental SEP (specifically education level and household income), results across both cohorts are consistent, thereby reinforcing their robustness. Our results show that parental lifestyle and BMI patterns during pregnancy mediate the inverse association between parents' education and child overweight. In ELFE, this relation is largely explained by suboptimal parental lifestyle behaviours during pregnancy (first pattern) beyond parental BMI status (second pattern). In Generation R, both patterns contributed equally to the mediation effect. Several hypotheses could explain why suboptimal parental lifestyle behaviours during pregnancy largely mediate the inverse association between parents' education and child overweight, one involving a greater health literacy of parents with high education attainment [36]. Such parents may better understand and thus implement public health recommendations. Enhanced comprehension and application could manifest in healthier lifestyle patterns adopted by both parents from pregnancy, potentially benefitting foetal development and metabolic programming. Furthermore, considering that parental lifestyle patterns tend to persist postpartum [41] and that parents serve as primary educators and role models for their child [42], children may likely adopt similar lifestyle habits, which in turn contribute to their energy balance equation related to overweight [43].

A Norwegian study [44] of 27 134 children aged 5 years used an inverse odds weighting approach and found a 63.7% mediating effect of prenatal and early postnatal factors on the relation between parents' education level and child overweight. A German study [45] of 4772 children aged 5 to 7 years showed a 77.8% mediation by parental lifestyle factors (BMI, smoking, shared meals, media consumption, physical activity) on this association, although these factors were not collected during pregnancy. These data support earlier findings, including a 2017 literature review [46] of factors influencing the SEP–adiposity association in youth up to age 18, highlighting parents' education level as a common SEP indicator and identifying consistent mediators such as breastfeeding duration, maternal pregnancy smoking, and infant feeding practices.

Although our analyses indicate a mediating effect of parents' lifestyle and BMI patterns on the association between household income level and child overweight in the ELFE but not Generation R children at age 5 years, they do not demonstrate a total effect for this association in either cohort. The absence of a total effect could be attributed to low statistical power as well as selection bias and differential attrition biases. These factors may have resulted in the initial selection of participants with high income levels and a greater loss of participants with low income levels over time, thus inhibiting our ability to include individuals with more precarious living conditions in our analyses. However, this variable should still be considered because household income during pregnancy reflects the family's financial capacity to access goods and services. All else being equal, increased income might enable access to wealthier neighbourhoods to live in and better products and services, such as more optimal prenatal care, healthier food options, and maternal health resources. These advantages could improve maternal nutrition and lifestyle patterns, positively influencing foetal development and metabolic programming of adipose tissue.

This study has some limitations. First, as already mentioned, as in all cohorts, there is an inherent selection and attrition bias, with individuals with high SEP more likely to participate and remain in the study than those experiencing socio‐economic disadvantage. This bias may potentially attenuate our ability to investigate the complete social gradient. The absolute value of the indirect effect may be even stronger with a better representation of underprivileged populations as well as more precise measurements of lifestyle behaviours during pregnancy (e.g., objective measurements instead of questionnaires) [47, 48]. Additionally, conducting the study in two distinct cohorts from different countries presented challenges in harmonisation of variables. Despite rigorous efforts to standardise variables, some differences remained across measures. For example, in the ELFE cohort, income was calculated per consumption unit, while in the Generation R cohort, total net household income was used, without adjustment for household size or composition. These methodological differences, combined with unequal purchasing power between countries, limit the comparability of income‐related analyses across cohorts. Another example is sedentary behaviour, which was measured in ELFE but not in Generation R and thus characterises the first lifestyle patterns in ELFE but not in Generation R, potentially affecting the comparability of results.

However, this double analysis, conducted both within ELFE and Generation R cohorts, is also a key strength, providing valuable insights across diverse settings. On the one hand, consistent findings (e.g., with education level) support their robustness. On the other, discrepant findings (e.g., with geographic background and income) highlight the importance of accounting for country‐specific factors when targeting at‐risk populations and tailoring public health interventions. Another strength is in examining both education level and income to approximate SEP because they capture a non‐interchangeable dimension of socio‐economic vulnerability [34], whereas most studies focus solely on education level. Furthermore, this study is unique in including the parents' geographic origin variable along with SEP, thus adding another dimension to these analyses by going beyond purely socio‐economic aspects. Finally, the analysis used principal component analysis to address lifestyle and BMI status as patterns rather than individual risk factors. This approach enables a more comprehensive and integrated examination of numerous variables that would otherwise be correlated and would have challenged the mediation analysis. It provides a holistic view that more accurately reflects real‐life scenarios and enhances our understanding of the complex interactions influencing health outcomes. It also takes into account paternal factors, in terms of social and health determinants and within a broader context of lifestyle patterns. These aspects are not usually included in similar analyses, which represents a key strength of this research in that it shifts the focus from the mother to the parental dyad.

Our study highlights the importance of addressing various dimensions of social vulnerability when developing multi‐behavioural interventions. Enhancing knowledge and health literacy with social support is crucial for empowering individuals to improve their health behaviours [34]. A growing body of evidence is emerging, demonstrating the beneficial impact of interventions targeting both parents on obesity risk and associated behaviours in children during the first 1000 days of life, especially those living with social disadvantage [49]. The lifestyle patterns identified during pregnancy are likely part of broader trajectories that begin before conception and extend into the postnatal period [50]. Our focus on prenatal exposures was motivated by the fact that parental lifestyles during pregnancy represent upstream determinants in the causal chain—likely shaping the postnatal family environment and, ultimately, the child's own behaviours. Nevertheless, further research into postnatal influences remains crucial, particularly to better understand the mechanisms underlying the association between parents' geographic origin and the risk of childhood overweight, which remains insufficiently explained by the prenatal period examined in this study.

## 5 Conclusion

A substantial part of the association between parents' education level and childhood overweight was attributable to suboptimal parental lifestyle and BMI patterns during pregnancy in both ELFE and Generation R cohorts, with the strength of this mediation varying between the cohorts. Conversely, these patterns did not explain the association between parents' geographic origin and childhood overweight. Mechanisms explaining this association seem to involve other unidentified factors. Our findings underscore the need for context‐specific public health strategies tailored to the socio‐economic and socio‐cultural characteristics of families as early as pregnancy to mitigate inequalities in childhood overweight.

## Author Contributions

C Le Gal, B Heude, S Lioret, MC Schipper and R Gaillard designed the research, wrote the manuscript and analysed the data. M Lecorguillé has provided data curation for this project. S Lioret, B Heude, MC Schipper, R Gaillard, M Lecorguillé, L Pavicic, T Simeon, MA Charles reviewed drafts and provided critical feedback. All authors approved the final manuscript and were responsible for the final content of the paper.

## Ethics Statement

The ELFE study received approvals from the Advisory Committee for the Processing of Information for Health Research (Comité Consultatif sur le Traitement des Informations pour la Recherche en Santé) and National Data Protection Authority (Commission National Informatique et Libertés) and the National Council for Statistical Information. All research was performed in accordance with the Declaration of Helsinki. Generation R study approval was obtained by the Medical Ethical Committee of the Erasmus Medical Center, University Medical Center, Rotterdam (MEC 198.782/2001/31).

## Consent

In ELFE study, consent for their child's participation was obtained from mothers, with fathers (if present) given the chance to consent and informed of their right to oppose the study. Recognising the language diversity among the population, study materials, including information letter and consent form, were made available in Arabic, Turkish, and English, the three most commonly spoken languages in France among non‐French speakers. In the Generation R study, mothers and fathers were asked for their written informed consent for the four consecutive phases of the study (prenatal, birth to 4 years, 4–16 years, and > 16 years). At the start of each phase, mothers and fathers received written and oral information about the study.

## Conflicts of Interest

The authors declare no conflicts of interest.

## Supporting information


**Data S1:** ijpo70047‐sup‐0001‐Supinfo.docx.

## Data Availability

The data that support the findings of this study are available from the corresponding author upon reasonable request.
