# Supplementary material for: Association Between Parental Social Position and Childhood Overweight: Mediation by Lifestyle and BMI Patterns During Pregnancy
Source: Pediatr Obes. 2025 Aug 5;20(11):e70047. doi: 10.1111/ijpo.70047 (PMC12501042; doi:10.1111/ijpo.70047)
Supplement: Supplementary file 1 — Data S1: ijpo70047‐sup‐0001‐Supinfo.docx. [file IJPO-20-e70047-s001.docx]

***Supplementary section***

**Parents’ geographic origin:**

The geographic origin of both parents was determined by the birth countries of their own parents (i.e., the child's grandparents). According to this framework, parents with both parents born in Western countries were classified as "Western", those with only one parent born outside a Western country as "mixed", and those with both parents born outside a Western country as "non-Western". For the sake of parsimony and simplicity in interpreting the mediation analyses, final variables were reclassified into binary groups: "no Non-Western parent” versus “at least one non-Western parent”. Further details regarding this classification and the specific countries included within the term “Western country” are elucidated in ***Supplemental Table 1***.

**
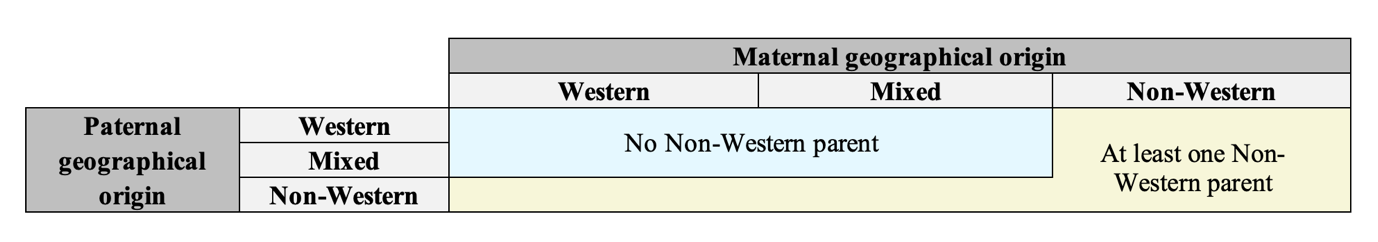
**

***Table 1.*** *Classification of parents’ geographic origin.*

**Western countries include European Union, Andorra, Australia, Canada, Iceland, Liechtenstein, Monaco, New Zealand, Norway, San Marino, Switzerland, USA and Vatican City. Non-western countries include all other countries.*

**Parents’ education level:**

Parents’ education was determined by the highest level achieved by either parent and classified as "≤ high school degree " versus "≥ undergraduate degree" (corresponding to short-cycle tertiary education or above), regardless of whether both parents lived in the same household. This approach was chosen to reflect the overall educational background of the parental dyad at the very beginning of life and is consistent with previous literature using similar indicators of SEP.

**
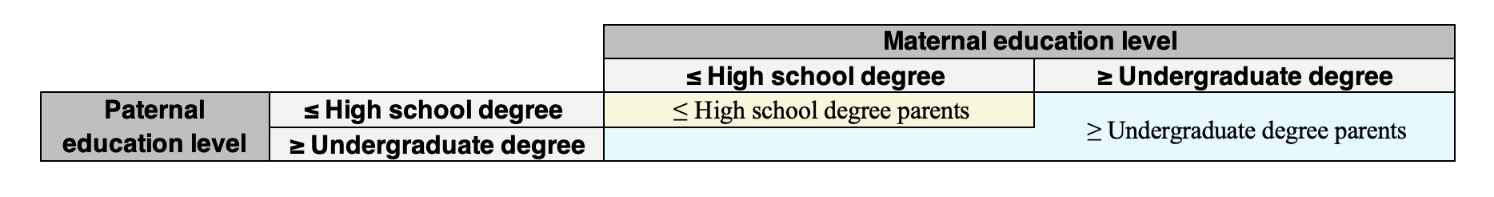
**

***Table 2.*** *Classification of parents’ education level.*

**Household income level:**

In the ELFE cohort, household income level per consumption unit was calculated according to the definition from the French National Institute of Statistics and Economic Studies by assigning different weights to household members based on age (1 unit for the primary household member, 0.5 for members aged ≥ 14 years, and 0.3 for each child < 14 years), thus facilitating a comparison of living standards across households of various sizes and compositions. The French variable was classified into income quintiles, with quintile 1 representing the lowest income level and quintile 5 representing the highest income level. In the Generation R cohort, net household income was used. Subsequently, income was dichotomized as "quintile 1" versus " quintile > 1" (ELFE) and “≤ 1200 euros” versus “>1200 euros” (Generation R). The category "<1200 euros" within Generation R closely approximates the lowest income quintile. However, it would not have been appropriate to select the exact same amount, which does not correspond to the same purchasing power in both countries.

STROBE Statement—Checklist of items that should be included in reports of ***cohort studies***

|  | Item No | Recommendation |  | Respected? |  |  |  | Comments and quotes |  |  |  |
| --- | --- | --- | --- | --- | --- | --- | --- | --- | --- | --- | --- |
| **Title and abstract** | 1 | (*a*) Indicate the study’s design with a commonly used term in the title or the abstract |  | Yes |  |  |  | The abstract provides a clear indication of the study's design. Quote: "the French national cohort Etude Longitudinale Française depuis l'Enfance (ELFE) (n=8584) and the Dutch Generation R birth cohort (n=6511)..."" |  |  |  |
|  |  | (*b*) Provide in the abstract an informative and balanced summary of what was done and what was found |  | Yes |  |  |  | The abstract provides a balanced summary of the study’s methodology, results, and conclusions. Quote: " We used counterfactual mediation analyses to assess the potential mediating effect..." |  |  |  |
| Introduction | | |  |  |  |  |  |  |  |  |  |
| Background/rationale | 2 | Explain the scientific background and rationale for the investigation being reported |  | Yes |  |  |  | Page 3, Lines 1-24 and Page 4, Lines 1-16 |  |  |  |
| Objectives | 3 | State specific objectives, including any prespecified hypotheses |  | Yes |  |  |  | Page 4, Lines 17-23 |  |  |  |
| Methods | | |  |  |  |  |  |  |  |  |  |
| Study design | 4 | Present key elements of study design early in the paper |  | Yes |  |  |  | Page 5, Lines 3-22 |  |  |  |
| Setting | 5 | Describe the setting, locations, and relevant dates, including periods of recruitment, exposure, follow-up, and data collection |  | Yes |  |  |  | Setting and Location: Page 5, Lines 3-5 and Lines 14-15  Relevant date including periods of recruitment: Page 5, Lines 5-6 and Lines 16  Exposure, follow-up and data collection: Page 5, Lines 24-26, Page 6, Lines 2-24 and Page 7, Lines 1-10 |  |  |  |
| Participants | 6 | (*a*) Give the eligibility criteria, and the sources and methods of selection of participants. Describe methods of follow-up |  | Yes |  |  |  | Page 5, Lines 4-21 |  |  |  |
|  |  | (*b*) For matched studies, give matching criteria and number of exposed and unexposed |  | N/A |  |  |  |  |  |  |  |
| Variables | 7 | Clearly define all outcomes, exposures, predictors, potential confounders, and effect modifiers. Give diagnostic criteria, if applicable |  | Yes |  |  |  | Page 5, Lines 23-26, Page 6, Lines 2-24 and Page 7, Lines 1-10 |  |  |  |
| Data sources/ measurement | 8* | For each variable of interest, give sources of data and details of methods of assessment (measurement). Describe comparability of assessment methods if there is more than one group |  | Yes |  |  |  | Page 5, Lines 24-26, Page 6, Lines 2-24 and Page 7, Lines 1-10 |  |  |  |
| Bias | 9 | Describe any efforts to address potential sources of bias |  | Yes |  |  |  | Adjustments: Page 8, Lines 20-25 and Page 9, Lines 1-3  Multivariate Imputation by Chained Equations: Page 9, Lines 4-11 |  |  |  |
| Study size | 10 | Explain how the study size was arrived at |  | N/A |  |  |  |  |  |  |  |
| Quantitative variables | 11 | Explain how quantitative variables were handled in the analyses. If applicable, describe which groupings were chosen and why |  | Yes |  |  |  | Page 7, Lines 23-25, Page 8, Lines 1-18 |  |  |  |
| Statistical methods | 12 | (*a*) Describe all statistical methods, including those used to control for confounding |  | Yes |  |  |  | Page 7, Lines 23-25, Page 8, Lines 1-25, Page 9 Lines1-3 |  |  |  |
|  |  | (*b*) Describe any methods used to examine subgroups and interactions |  | N/A |  |  |  |  |  |  |  |
|  |  | (*c*) Explain how missing data were addressed |  | Yes |  |  |  | Multivariate Imputation by Chained Equations: Page 9, Lines 4-11 |  |  |  |
|  |  | (*d*) If applicable, explain how loss to follow-up was addressed |  | N/A |  |  |  |  |  |  |  |
|  |  | (*e*) Describe any sensitivity analyses |  | N/A |  |  |  |  |  |  |  |
| Results | | |  |  |  |  |  |  |  |  |  |
| Participants | 13* | (a) Report numbers of individuals at each stage of study—eg numbers potentially eligible, examined for eligibility, confirmed eligible, included in the study, completing follow-up, and analysed |  | Yes |  |  |  | Page 7, Lines 12-20 |  |  |  |
|  |  | (b) Give reasons for non-participation at each stage |  | Yes |  |  |  | Flow Chart in Figure 1 |  |  |  |
|  |  | (c) Consider use of a flow diagram |  | Yes |  |  |  | Flow Chart in Figure 1 |  |  |  |
| Descriptive data | 14* | (a) Give characteristics of study participants (eg demographic, clinical, social) and information on exposures and potential confounders |  |  |  |  |  |  |  |  |  |
|  |  | (b) Indicate number of participants with missing data for each variable of interest |  | Yes |  |  |  | Table 1 |  |  |  |
|  |  | (c) Summarise follow-up time (eg, average and total amount) |  | Yes |  |  |  | Page 7, Lines 16-17 |  |  |  |
| Outcome data | 15* | Report numbers of outcome events or summary measures over time |  | N/A |  |  |  |  |  |  |  |
| Main results | 16 | (*a*) Give unadjusted estimates and, if applicable, confounder-adjusted estimates and their precision (eg, 95% confidence interval). Make clear which confounders were adjusted for and why they were included |  | N/A |  |  |  |  |  |  |  |
|  |  | (*b*) Report category boundaries when continuous variables were categorized |  | N/A |  |  |  |  |  |  |  |
|  |  | (*c*) If relevant, consider translating estimates of relative risk into absolute risk for a meaningful time period |  | N/A |  |  |  |  |  |  |  |
| Other analyses | 17 | Report other analyses done—eg analyses of subgroups and interactions, and sensitivity analyses |  | N/A |  |  |  |  |  |  |  |
| Discussion | | |  |  |  |  |  |  |  |  |  |
| Key results | 18 | Summarise key results with reference to study objectives |  | Yes |  |  |  | Page 11, Lines 2-8 |  |  |  |
| Limitations | 19 | Discuss limitations of the study, taking into account sources of potential bias or imprecision. Discuss both direction and magnitude of any potential bias |  | Yes |  |  |  | Page 14, Lines 14-25 |  |  |  |
| Interpretation | 20 | Give a cautious overall interpretation of results considering objectives, limitations, multiplicity of analyses, results from similar studies, and other relevant evidence |  | Yes |  |  |  | Page 11, Lines 21-25, Page 12, Lines 1-25, Page 13, Lines 1-25 and Page 14, Lines 1-9 |  |  |  |
| Generalisability | 21 | Discuss the generalisability (external validity) of the study results |  | Yes |  |  |  | Page 15, Lines 20-25 and Page 16, Lines 1-8 |  |  |  |
| Other information | | |  |  |  |  |  |  |  |  |  |
| Funding | 22 | Give the source of funding and the role of the funders for the present study and, if applicable, for the original study on which the present article is based |  | Yes |  |  |  | Page 21, Lines 13-25 and Page 22, Lines 1-13 |  |  |  |

*Give information separately for exposed and unexposed groups.

**Note:** An Explanation and Elaboration article discusses each checklist item and gives methodological background and published examples of transparent reporting. The STROBE checklist is best used in conjunction with this article (freely available on the Web sites of PLoS Medicine at http://www.plosmedicine.org/, Annals of Internal Medicine at http://www.annals.org/, and Epidemiology at http://www.epidem.com/). Information on the STROBE Initiative is available at http://www.strobe-statement.org.
